# A combined approach for comparative exoproteome analysis of *Corynebacterium pseudotuberculosis*

**DOI:** 10.1186/1471-2180-11-12

**Published:** 2011-01-17

**Authors:** Luis GC Pacheco, Susan E Slade, Núbia Seyffert, Anderson R Santos, Thiago LP Castro, Wanderson M Silva, Agenor V Santos, Simone G Santos, Luiz M Farias, Maria AR Carvalho, Adriano MC Pimenta, Roberto Meyer, Artur Silva, James H Scrivens, Sérgio C Oliveira, Anderson Miyoshi, Christopher G Dowson, Vasco Azevedo

**Affiliations:** 1 Department of Biochemistry and Immunology, Instituto de Ciências Biológicas, Universidade Federal de Minas Gerais, Av. Antônio Carlos, Belo Horizonte, 31.270-901, Brazil; 2 Department of General Biology, Instituto de Ciências Biológicas, Universidade Federal de Minas Gerais, Av. Antônio Carlos, Belo Horizonte, 31.270-901, Brazil; 3 Institute of Health Sciences, Universidade Federal da Bahia, Av. Reitor Miguel Calmon, Salvador, 40.110-902, Brazil; 4 School of Life Sciences, University of Warwick, Gibbet Hill Road, Coventry, CV4 7AL, United Kingdom; 5 Department of Microbiology, Instituto de Ciências Biológicas, Universidade Federal de Minas Gerais, Av. Antônio Carlos, Belo Horizonte, 31.270-901, Brazil; 6 Genome and Proteome Network of the State of Pará, Universidade Federal do Pará, R. Augusto Corrêa, Belém, 66.075-110, Brazil

## Abstract

**Background:**

Bacterial exported proteins represent key components of the host-pathogen interplay. Hence, we sought to implement a combined approach for characterizing the entire exoproteome of the pathogenic bacterium *Corynebacterium pseudotuberculosis*, the etiological agent of caseous lymphadenitis (CLA) in sheep and goats.

**Results:**

An optimized protocol of three-phase partitioning (TPP) was used to obtain the *C. pseudotuberculosis *exoproteins, and a newly introduced method of data-independent MS acquisition (LC-MS^E^) was employed for protein identification and label-free quantification. Additionally, the recently developed tool SurfG+ was used for *in silico *prediction of sub-cellular localization of the identified proteins. In total, 93 different extracellular proteins of *C. pseudotuberculosis *were identified with high confidence by this strategy; 44 proteins were commonly identified in two different strains, isolated from distinct hosts, then composing a core *C. pseudotuberculosis *exoproteome. Analysis with the SurfG+ tool showed that more than 75% (70/93) of the identified proteins could be predicted as containing signals for active exportation. Moreover, evidence could be found for probable non-classical export of most of the remaining proteins.

**Conclusions:**

Comparative analyses of the exoproteomes of two *C. pseudotuberculosis *strains, in addition to comparison with other experimentally determined corynebacterial exoproteomes, were helpful to gain novel insights into the contribution of the exported proteins in the virulence of this bacterium. The results presented here compose the most comprehensive coverage of the exoproteome of a corynebacterial species so far.

## Background

*Corynebacterium pseudotuberculosis *is a facultative intracellular pathogen that belongs to the so-called CMN (*Corynebacterium-Mycobacterium-Nocardia*) group, a distinct subgroup of the *Actinobacteria *that also includes other highly important bacterial pathogens, such as *Corynebacterium diphtheriae *and *Mycobacterium tuberculosis*. The most distinctive feature of these Gram-positive bacteria is the unique composition of the cell envelope, characterized by the presence of long chain fatty acids, known as mycolic acids, on the surface of the cell [1,2].

The main recognizable disease caused by *C. pseudotuberculosis *is caseous lymphadenitis (CLA) in sheep and goats, though this bacterium can also infect several other hosts, including humans [1,3]. Typical manifestations of CLA in small ruminants include formation of abscesses in superficial and internal lymph nodes, and in visceral organs [3]. Despite the important economic losses caused by this disease to sheep and goat husbandry worldwide, no effective treatment exists, and the efficacy of the currently available vaccines and diagnostic methods is still controversial [4].

The search for *C. pseudotuberculosis *molecular determinants that contribute to CLA pathogenesis lead to the recognition of two exported proteins as the major virulence-associated factors of this bacterium known to date: a secreted phospholipase D (PLD) [5]; and an ABC-type transporter component of an iron uptake system (FagB) [6]. In fact, one might expect that the majority of the virulence determinants of *C. pseudotuberculosis *would be present in the exoproteome, *i.e*. the entire set of bacterial proteins found in the extracellular milieu [7]. This is because exported proteins participate in essential steps of the host-pathogen interplay, including: (i) adhesion to host cells; (ii) invasion; (iii) damage to host tissues; (iv) resistance to environmental stresses during infection; and (iv) subversion of the host's immune response mechanisms [8-10].

In two previous attempts to characterize the *C. pseudotuberculosis *exoproteome, our group optimized a protocol of salting out of proteins using sulfate and butanol, known as three-phase partitioning (TPP), for isolation of the extracellular proteins of this bacterium [11], and generated a library of *C. pseudotuberculosis *mutant strains possessing transposon insertions in genes coding for probable exported proteins [12]. In the former study, we were able to determine the optimal conditions for obtaining the best recovery of immunoreactive extracellular proteins of *C. pseudotuberculosis *[11]. The second study in turn, enabled us to identify various previously uncharacterized *C. pseudotuberculosis *exported proteins, being that at least two of them are apparently involved in virulence [12]. Now, the very recent conclusion of the *C. pseudotuberculosis *Genome Project by our group, associated to the current availability of high-throughput proteomic technologies, permitted us to perform a much more comprehensive analysis of this bacterium's exoproteome.

In this study, we sought to implement a combined approach for comparative exoproteome analysis of different *C. pseudotuberculosis *strains. The strategy included: (i) the previously optimized TPP protocol for isolation of the extracellular proteins [11]; (ii) a newly introduced method of data-independent LC-MS acquisition (LC-MS^E^) for protein identification and quantification [13,14]; and (iii) the recently developed tool SurfG+ for *in silico *prediction of protein sub-cellular localization in Gram-positive bacteria [15]. We believe that the experimental approach used is very suitable for profiling bacterial exoproteomes, as it shown to be easily applicable to different strains with very good reproducibility. This is an advantage over what is commonly observed for proteomic approaches based on two-dimensional (2D) gel electrophoresis, where there is more variability, but is apparently the method of choice for most of the bacterial exoproteome studies published recently [16-20]. Furthermore, the LC-MS^E ^method provides high subproteome coverage, due to enhanced sensitivity, and allows for label-free analysis of differentially expressed proteins [14]; this latter possibility enables the detection of variations in the exoproteomes of different strains that could be missed by simply profiling the exoproteins, and meets the growing interest in performing physiological proteomic studies of bacteria [21,22].

We were able to identify 93 different *C. pseudotuberculosis *extracellular proteins with high confidence by analyzing the exoproteomes of two strains isolated from different hosts that presented distinct virulence phenotypes under laboratory conditions [23,24]. Most of the identified proteins were predicted *in silico *to have an extracytoplasmic localization. To the best of our knowledge, these results compose the largest inventory of experimentally confirmed exoproteins of a single corynebacterial species to date. Importantly, the comparative exoproteome analyses permitted us to speculate on the probable contributions of different *C. pseudotuberculosis *extracellular proteins to the virulence of this bacterium.

## Results and Discussion

### Exoproteome analysis of *Corynebacterium pseudotuberculosis*

The extracellular proteins of two *C. pseudotuberculosis *strains, one isolated from a goat (strain 1002) the other from a sheep (strain C231), cultivated in a chemically-defined medium, were extracted/concentrated by the TPP technique. The trypsinized protein samples were then submitted to LC-MS^E ^analysis.

**Figure 1:**
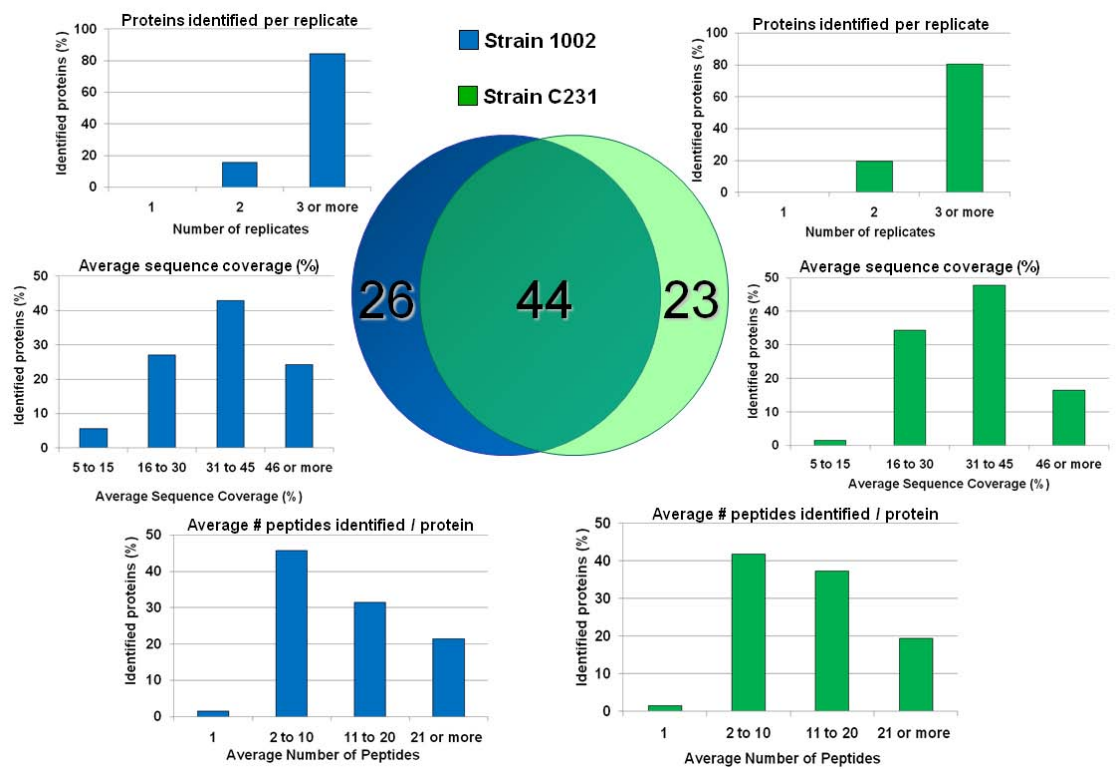
**Analysis of the extracellular proteins of two different *C. pseudotuberculosis *strains allowed for identification of the core and variant exoproteomes**. TPP-extracted extracellular proteins of the strains 1002 and C231 of *C. pseudotuberculosis *were submitted to LC-MS^E ^analysis. The Venn-diagram shows the numbers of commonly identified and variant exoproteins between the strains. The number of replicates in which a given protein was observed, the average peptides identified per protein, and the average sequence coverage of the proteins in each exoproteome studied, are shown as frequency distributions for comparison purposes.

Seventy soluble extracellular proteins of the 1002 strain could be confidentially identified by this methodology, whereas the number of proteins identified in the exoproteome of the C231 strain was sixty-seven. Altogether, 93 different *C. pseudotuberculosis *exoproteins were identified in this study (Figure 1). These findings agree with the results of previous experiments by our group, in which we have used a 2D-PAGE based strategy for a preliminary appraisal of the *C. pseudotuberculosis *exoproteome (additional file 1). Eighty protein spots, mostly concentrated in the pI range between 3.0 and 6.0, could be reproducibly visible in the 2D gels generated from TPP-extracted extracellular proteins of the 1002 strain (additional file 1). The fact that we have found 70 proteins in the exoproteome of this strain with high confidence when using the LC-MS^E ^method (Figure 1) indicates that this novel methodology allowed us to identify virtually the complete set of extracellular proteins that are commonly observed in the gel based methodologies (additional file 1). Moreover, the expected existence of protein isoforms among the eighty protein spots observed in the 2D gels, and the identification by LC-MS^E ^of many proteins out of the pI range 3.0-6.0, suggests that the latter methodology is much more suitable for obtaining a comprehensive coverage of the bacterial exoproteome. Noteworthy, is the use of LC-MS^E ^for exoproteome profiling which required (i) much less time and labor than the gel based proteomic strategy, and (ii) much less protein sample necessary for each experimental replicate, with only 0.5 μg per replicate used in the LC-MS^E ^compared to 150 μg for the 2D gels [refer to Patel *et al*. [25] for a comprehensive comparison on these proteomic strategies].

The performance of the combined methodology used in the present study (TPP/LC-MS^E^) for mapping the *C. pseudotuberculosis *exoproteome was very similar for both strains analyzed, as can be seen by the average numbers of peptides observed per protein in the two proteomes (16.5 and 15.0) and by the average sequence coverage of the proteins identified (37.5% and 35.0%) (Figure 1). Consistent with this, the majority of the proteins detected in each extracellular proteome were shared by the goat and sheep isolates; this permitted us to define a core *C. pseudotuberculosis *exoproteome composed of 44 proteins out of the 93 different extracellular proteins identified. Additional files 2, 3 and 4 list all the proteins identified in the exoproteomes of the two *C. pseudotuberculosis *strains, along with molecular weights, isoelectric points, main orthologs, predicted sub-cellular localizations, number of peptides experimentally observed, and sequence coverage.

Searches of similarity against publicly available protein databases using the Blast-p tool [26] showed that ortholog proteins can be found in the pathogenic *Corynebacterium diphtheriae *for most of the identified *C. pseudotuberculosis *exoproteins (additional files 2, 3 and 4), as would be expected due to the close phylogenetic relationship of these species [27]. Nevertheless, no significant orthologs could be found for six proteins of the *C. pseudotuberculosis *exoproteome, even when using the position-specific iterated BLAST (PSI-BLAST) algorithm [28], namely the proteins [GenBank:ADL09626], [GenBank:ADL21925], [GenBank:ADL11253], [GenBank:ADL20222], [GenBank:ADL09871], and [GenBank:ADL21537] (additional files 2, 3 and 4). With the exception of [GenBank:ADL11253], all these proteins were predicted by different tools as being truly exported proteins. This means they are the only five exoproteins identified in this study which are probably unique for *C. pseudotuberculosis*.

### Prediction of sub-cellular localization of the identified proteins

**Figure 2:**
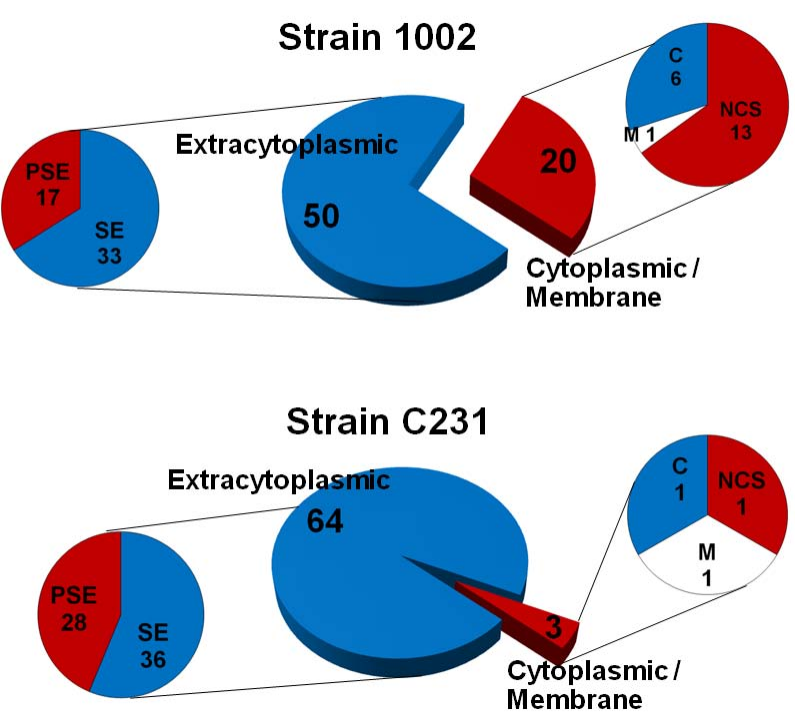
**Most of the identified *C. pseudotuberculosis *exoproteins were predicted by the SurfG+ program as having an extracytoplasmic localization**. The proteins identified in the exoproteomes of each *C. pseudotuberculosis *strain were analyzed by SurfG+ and attributed a probable final sub-cellular localization. Proteins classified as having a cytoplasmic localization were further analyzed with the SecretomeP tool for prediction of non-classical (leaderless) secretion. Besides, literature evidence for exportation by non-classical pathways was also used to re-classify the cytoplasmic proteins (see text for details). SE = secreted; PSE = potentially surface exposed; C = cytoplasmic; M = membrane; NCS = non-classically secreted.

Most of the proteins identified in the exoproteomes of the two *C. pseudotuberculosis *strains were also predicted to have a probable extracytoplasmic localization after *in silico *analysis of the sequences of these proteins with different bioinformatics tools, thereby corroborating our *in vitro *findings (Figure 2, additional file 5). It is important to note here that we are considering the exoproteome as the entire set of proteins released by the bacteria into the extracellular milieu. That means we are looking to: (i) proteins possessing classical signals for active exportation by the different known mechanisms, which are directly secreted into the cell supernatant or that remain exposed in the bacterial cell surface and are eventually released in the growth medium [7]; and (ii) proteins exported by non-classical pathways, without recognizable signal peptides [29]. Besides, one might also expect to observe in the extracellular proteome a small number of proteins primarily known to have cytoplasmic localization; although some of these proteins are believed to be originated from cell lysis or leakage, like in the extreme situation reported by Mastronunzio *et al*. [19], a growing body of evidence suggests that moonlighting proteins (in this case, cytoplasmic proteins that assume diverse functions in the extracellular space) may be commonly found in the bacterial exoproteomes [29-32].

By using the recently developed tool SurfG+ we were able to classify the identified *C. pseudotuberculosis *proteins into four different categories: (i) secreted, (ii) potentially surface exposed (PSE), (iii) membrane and (iv) cytoplasmic (Figure 2, additional files 2, 3 and 4). Basically, this software brings together the predictions of global protein localizations performed by a series of well-known algorithms, and innovates by allowing for an accurate prediction of PSE proteins [15]. This possibility of classification provides us with valuable information on the proteins identified, as bacterial surface exposed proteins are believed to play important roles in the host-pathogen interactions during infection and many of these proteins have been shown to be highly protective when used in vaccine preparations [33,34].

From a total of 93 different *C. pseudotuberculosis *proteins identified in this study, 75% (70) could be predicted as containing signals for active exportation (secretion or surface exposition) following SurfG+ analysis (Figure 2). Taken together, these proteins represent roughly 50% of all predicted secreted proteins in the recently sequenced genome of *C. pseudotuberculosis*, and around 15% of all predicted PSE proteins of this bacterium (A.R. Santos, pers. comm.).

The concordance of our *in vitro *identification of exoproteins with the *in silico *predictions of protein exportation is higher than what has normally been observed in recent exoproteome analyses of different bacteria [17-19,35,36]. For comparison, Hansmeier *et al*. [17] reported that exportation signals could be predicted in only 42 (50%) out of 85 different proteins identified in the extracellular and cell surface proteomes of *Corynebacterium diphtheriae*. The authors of this study are not the only to speculate on a probably important contribution of cross-contamination of the protein sample during preparation procedures for the observation of high numbers of proteins not predicted as having extracellular location in the bacterial exoproteomes [17,31]. We believe that the proportionally higher identification of proteins possessing exportation signals in the present study could have happened due to a series of different factors, including: (i) our methodology for isolation of the bacterial extracellular proteins might have extracted less "contaminant" cytoplasmic proteins than did other methodologies reported in previous studies; (ii) the combined strategy used by SurfG+ to predict protein sub-cellular localization might have performed better in the identification of exported proteins than happened with other strategies, sometimes based in only one prediction tool; (iii) the fact that we have included in the final exoproteome lists only proteins identified with high confidence, in at least two experimental replicates, reduced significantly the possibilities of false-positive identifications that might account for some of the unexpected proteins; and finally (iv) the lower proportion of proteins primarily regarded as cytoplasmic might be actually a typical characteristic of the *C. pseudotuberculosis *exoproteome.

### Non-classically secreted proteins

Intriguingly, a much higher proportion (29.0%) of the exoproteome of the 1002 strain of *C. pseudotuberculosis *was composed by proteins predicted by SurfG+ as not having an extracytoplasmic location, when compared to only 4.5% in the exoproteome of the strain C231 (Figure 2). The possibility of these proteins being non-classically secreted has been evaluated using the SecretomeP algorithm [29]. We have also reviewed the literature for evidence of other bacterial exoproteomes that could support the extracellular localization found for these proteins in our study.

High SecP scores (above 0.5) could be predicted for 5 of the 19 proteins in the exoproteome of the 1002 strain considered by SurfG+ as having a cytoplasmic location (additional files 2 and 3); this could be an indicative that they are actually being secreted by non-classical mechanisms [29]. Nonetheless, 2 of these 5 proteins ([GenBank:ADL09626] and [GenBank:ADL20555]) were also detected in the exoproteome of the C231 strain, in which they were predicted by SurfG+ as possessing an extracytoplasmic location (additional file 2). A comparative analysis of the sequences encoding these proteins in the genomes of the two *C. pseudotuberculosis *strains showed that the disparate results were generated due to the existence of nonsense mutations in the genome sequence of the 1002 strain, which impaired the identification of signal peptides for the two proteins at the time of SurfG+ analysis (data not shown). We believe that it is unlikely that these differences represent true polymorphisms, as the proteins were identified in the extracellular proteome, indicating the real existence of exportation signals. This indeed demonstrates the obvious vulnerability of the prediction tools to the proper annotation of the bacterial genomes. On the other hand, the assignment of high SecP scores to these two proteins, even though they are not believed to be secreted by non-classical mechanisms, would be totally expected, as the SecretomeP is a predictor based on a neural network trained to identify general features of extracellular proteins; this means the prediction tool will attribute SecP scores higher than 0.5 to most of the secreted proteins, regardless the route of export [29].

We have found reports in the literature that strongly support the extracellular localization observed for 8 of the 14 remaining proteins considered as non-secretory by SurfG+ and SecretomeP in the exoproteome of the 1002 strain, and without any detectable signal peptide (additional files 2 and 3, Figure 2). Among these proteins there are the elongation factors Tu and Ts [16,33,35,37-39]; the glycolytic enzymes triosephosphate isomerase, phosphoglycerate kinase and phosphoglycerate mutase [16-20,37-40]; the chaperonin GroES [16-18,20,39]; a putative peptidyl prolyl cis trans isomerase [17,18,35,37,41]; and a hydroperoxide reductase enzyme [17,35,39].

Proteins primarily regarded as cytoplasmic have consistently been identified in the exoproteomes of different bacterial species, and moonlighting roles in the extracellular environment have already been demonstrated for some of them [31,32], including evasion of host's immune system [42], adhesion to host cells [43,44], folding of extracytoplasmic proteins [41,45], and interaction between microorganisms [40,46]. Noteworthy, specific evidences for active secretion of such cytoplasmic proteins have been demonstrated for only a few examples to date, and demonstration of an extracellular function is still missing for many of these proteins [30,31].

### The variant exoproteome may account for differential virulence of the two *C. pseudotuberculosis *strains

A considerable number (49/93) of the extracellular proteins identified in this work was observed in only one of the two strains studied, then composing a variant experimental *C. pseudotuberculosis *exoproteome (additional files 3 and 4). Highly variant exoproteomes have also been reported recently for other Gram+ bacterial pathogens [20,36,39,47-49], and such a variation may be considered an important factor leading to the observable phenotypic dissimilarities and ultimately to differential virulence of the various strains [50,51]. Hecker *et al*. [36] reported on how the composition of the exoproteome can vary extremely within a single species, *Staphylococcus aureus*, being that only 7 out of 63 identified extracellular proteins were found in all the twenty-five clinical isolates studied.

One of the most intriguing results in the present study was the detection of the phospholipase D (PLD) protein only in the extracellular proteome of the strain C231 (additional file 4). As the regulation of PLD expression was demonstrated to be complex and highly affected by multiple environmental factors [52], we sought to detect this protein in the culture supernatant of the *C. pseudotuberculosis *1002 strain grown in a rich medium (brain-heart infusion broth) instead of only chemically-defined medium (CDM), but these attempts were also unfruitful (data not shown). Besides, we were not able to detect secretion of PLD following total exoproteome analysis of the 1002 strain grown under specific stress generating conditions (Pacheco *et al*., unpublished). The results strongly indicate that this protein is actually not being secreted by the 1002 strain in culture.

PLD is an exotoxin considered as the major virulence factor of *C. pseudotuberculosis *[5,52]. It possesses sphingomyelinase activity that contributes to endothelial permeability and then to spreading of the bacteria within the host [5]. Mutation of the *pld *gene in *C. pseudotuberculosis *rendered strains no longer capable of causing caseous lymphadenitis (CLA) in sheep and goats; the potential of these strains to be used as live attenuated vaccines was already evaluated [53-55]. Similarly, the strain 1002 of *C. pseudotuberculosis *was already tested as a possible live attenuated vaccine against CLA due to its natural low virulent status, and administration of this bacterium to goats did not cause lesions formation [23,56]. The molecular mechanisms leading to the low virulence of the 1002 strain however remain undetermined so far. We believe that non-secretion of PLD might be one of the main factors responsible for the lowered virulence of the strain. Importantly, we currently cannot affirm that the 1002 strain does not produce this protein while infecting a mammalian host. Besides, this strain still retains the capability of causing localized abscesses and disease in susceptible mice (Pacheco *et al*., unpublished results).

Other proteins believed to be associated with the virulence of *C. pseudotuberculosis *were also identified exclusively in the exoproteome of the C231 strain, namely FagD and Cp40 (Table 1). The former protein is a component of an iron uptake system, whose coding sequences are clustered immediately downstream of the *pld *gene in the *C. pseudotuberculosis *genome [6]. The latter protein is a secreted serine protease shown to be protective against CLA when used to vaccinate sheep [57].

**Table 1:** Formerly and newly identified^‡ ^exported proteins that may be associated with the virulence phenotype of *Corynebacterium pseudotuberculosis *strains

Protein Description^a^	GenBank Accession	Identified in the exoproteome of the strain^b^:		Orhologs found in other Corynebacteria^c^:		References
			
		1002	C231	Pathogenic	Non-pathogenic	
Phospholipase D (PLD)	ADL09524.1	No	Yes	Yes	No	[54]
Iron siderophore binding protein (FagD)	ADL09528.1	No	Yes	Yes	Yes	[6]
Serine proteinase precursor (CP40)	ADL11339.1	No	Yes	No	No	[57]

Putative iron transport system binding (secreted) protein	ADL10460.1	No	Yes	Yes	No	[12]
Glycerophosphoryl diester phosphodiesterase	ADL11410.1	No	Yes	Yes	No	This work. [72]
Putative surface-anchored membrane protein	ADL20074.1	Yes	Yes	Yes	No	This work.
Putative hydrolase (lysozyme-like)	ADL20788.1	Yes	Yes	Yes	No	This work.
Putative secreted protein	ADL21714.1	Yes	Yes	Yes	No	This work.
Putative sugar-binding secreted protein	ADL09872.1	No	Yes	Yes	No	This work.

^‡ ^The inclusion criteria followed three main requisites: (i) experimental detection of the proteins in the exoproteomes of the pathogenic *C. diphtheriae *and *C. jeikeium*; (ii) non-detection of the proteins in the exoproteomes of the non-pathogenic *C. glutamicum *and *C. efficiens*; and (iii) *in silico *detection of ortholog proteins in pathogenic, but not in non-pathogenic, corynebacteria through search of similarity against public protein repositories.

^a ^This protein list is not meant to be all-inclusive. Rather, it wants to give an overview of the exported proteins identified in this study for which it was possible to speculate on a probable involvement in *C. pseudotuberculosis *virulence after comparative proteomic analyses.

^b ^Proteins identified in this study by TPP/LC-MS^E^

^c ^Searches of similarity against publicly available protein databases using Blast-p.

Strikingly, one variant protein of the *C. pseudotuberculosis *exoproteome, a conserved hypothetical exported protein with a cutinase domain [GenBank:ADL10384], has its coding sequence present in the genome of the C231 strain but absent from the genome of the 1002 strain (additional file 6). The genomic structure of the gene's surroundings is indicative of a region prone to recombination events, such as horizontal gene transfer [58]. In fact, it seems that gene gain and loss are frequent events leading to variations observed in the bacterial exoproteomes [39,59].

### Variation of the core exoproteome: differential expression analysis of the common proteins by LC-MS^E^

In addition to identifying qualitative variations in the exoproteomes of the two *C. pseudotuberculosis *strains, we were also able to detect relative differences in expression of the proteins common to the two proteomes through label-free protein quantification by the LC-MS^E ^method. Relative protein quantification by this method can be obtained with basis on the accurate precursor ion mass and electrospray intensity data, acquired during the low energy scan step of the alternating scan mode of MS acquisition [14]. Importantly, this quantitative attribute of the technique opens up new possibilities of utilization, as grows the interest on the so-called physiological proteomics [21].

**Figure 3:**
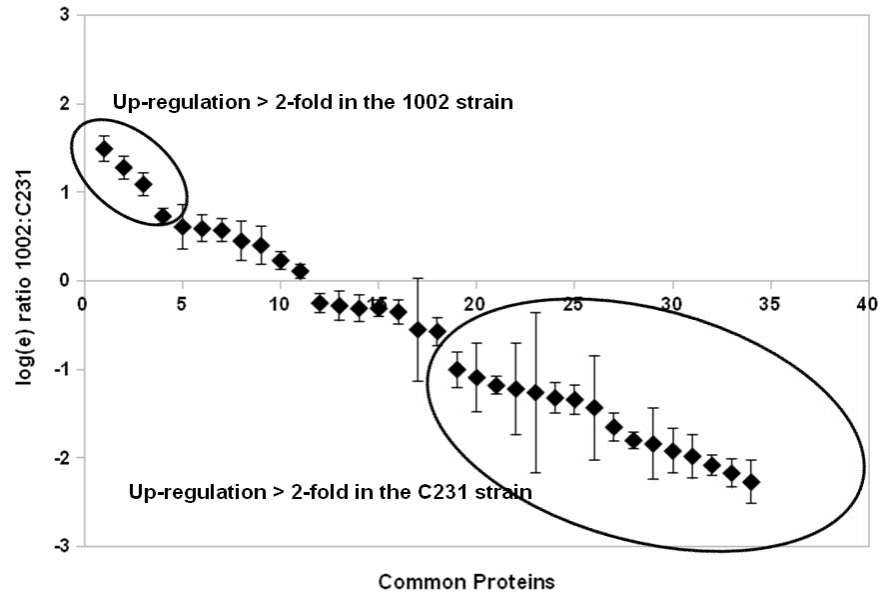
**Differential expression of the proteins composing the core *C. pseudotuberculosis *exoproteome, evaluated by label-free relative quantification using LC-MS^E^**. Results are shown as natural log scale of the relative quantifications (1002:C231) for each protein. Only proteins that were given a variation score higher than 250 by PLGS quantification algorithm are presented. Proteins regulated more than 2-fold in each strain are indicated. Protein identification numbers correspond to additional files 2 and 7: Tables S1 and S4.

Thirty-four out of 44 proteins commonly identified in the exoproteomes of the strains 1002 and C231 of *C. pseudotuberculosis *were considered by the PLGS quantification algorithm as having significantly variable expression (score > 250; 95% CI) (Figure 3, additional files 2 and 7). If we further filter these results for the proteins presenting differential expression higher than 2-fold between the strains, we end up with only four proteins up-regulated in the 1002 strain and sixteen in the C231 strain (Figure 3).

Among the group of proteins not presenting considerable variations in expression between the two *C. pseudotuberculosis *strains, proteins probably participating in basic bacterial physiological processes could be easily identified, as would be expected, including cell shape maintenance and cell division (penicillin binding protein, transglycosylases, peptidases, PGRP amidase) [60]; and iron uptake and utilization (HmuT) [61] (Figure 3, additional file 2). In this sense, one might also speculate that the hypothetical proteins identified as non variant in the two strains may have functions associated to the general physiology of *C. pseudotuberculosis*, when grown in minimal medium.

The most up-regulated proteins were observed in the extracellular proteome of the C231 strain, including two cell envelope-associated proteins [62], namely the major secreted (mycoloyltransferase) protein PS1 (10-fold up-regulated), and the S-layer protein A (8-fold up-regulation) (Figure 3). This may be indicative of differences on cell envelope-related activities in the two *C. pseudotuberculosis *strains, such as nutrient acquisition, protein export, adherence and interaction with the host [63]. Dumas *et al*. [49] compared the exoproteomes of *Listeria monocytogenes *strains of different virulence groups, and found that altered expression (up- or down-regulation) of a protein related to the bacterial cell wall could be a marker of specific virulence phenotypes. Additionally, surface associated proteins have been shown to undergo phase and antigenic variation in some bacterial pathogens, and ultimately affect the infectivity potential of different strains [50].

### Comparative analyses of corynebacterial exoproteomes

**Figure 4:**
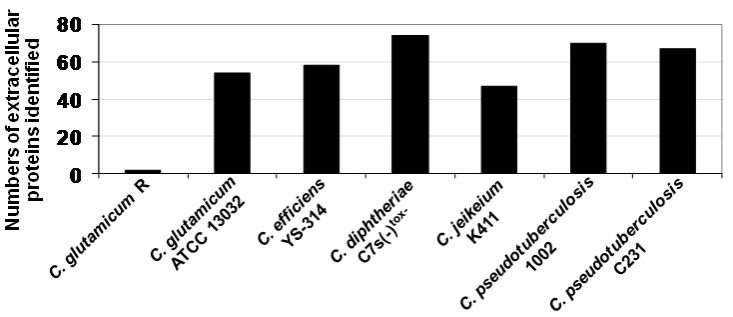
**Comparative analysis of corynebacterial exoproteomes**. Numbers of extracellular proteins identified in previous corynebacterial exoproteome analyses [17,37,69,70] in comparison to those identified in this study with the two strains of *C. pseudotuberculosis*.

Recent studies attempted to characterize the extracellular proteomes of other pathogenic (*C. diphtheriae *and *C. jeikeium*) and non-pathogenic (*C. glutamicum *and *C. efficiens*) corynebacterial species [17,37,64,65]. All these studies used 2D-PAGE to resolve the extracellular proteins of the different corynebacteria, and PMF by MALDI-TOF-MS was the method of choice in most of them for protein identification [17,37,64,65]. Figure 4 shows the numbers of proteins identified in the exoproteomes of all strains studied, in comparison to the numbers obtained in the present study for *C. pseudotuberculosis*. Despite one study with the strain R of *C. glutamicum*, which reports identification of only two secreted proteins [65], all the corynebacterial strains had somehow similar numbers of extracellular proteins identified, ranging from forty-seven in *C. jeikeium *K411 to seventy-four in *C. diphtheriae *C7s(-)^tox-^. Importantly, the fact that we have identified in this study 93 different exoproteins of *C. pseudotuberculosis*, through the analysis of two different strains, means that our dataset represents the most comprehensive exoproteome analysis of a corynebacterial species so far.

Regardless the different methodologies employed to characterize the exoproteomes of the various corynebacteria, we sought to identify extracellular proteins commonly identified in most of the studies, taking the catalogue of *C. pseudotuberculosis *exoproteins generated in this work as the comparison dataset. Besides corroborating our findings, the objective here was to identify extracellular proteins that could be associated exclusively to pathogenic corynebacterial species.

**Figure 5:**
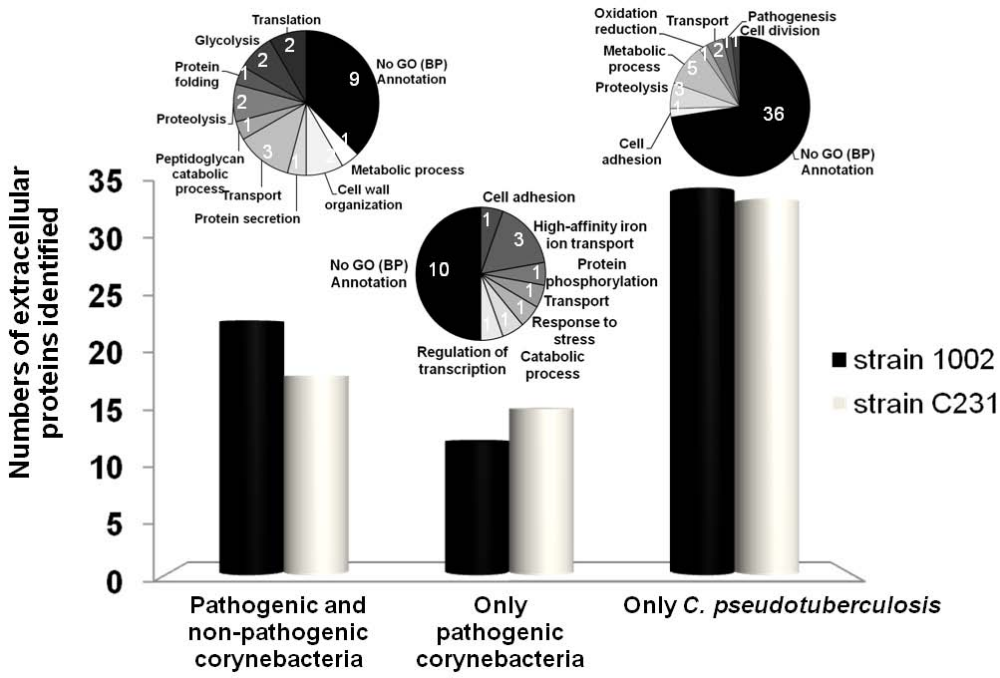
**Distribution of orthologous proteins of the *C. pseudotuberculosis *experimental exoproteins throughout other experimentally confirmed corynebacterial exoproteomes**. Pathogenic species: *C. diphtheriae *C7s(-)^tox- ^and *C. jeikeium *K411 [17,69]; non-pathogenic species: *C. glutamicum *ATCC13032 and *C. efficiens *YS-314 [37,70]. Pie charts show Gene Ontology (GO) functional annotations for the 93 different *C. pseudotuberculosis *exoproteins identified (24 commonly identified in pathogenic and non-pathogenic corynebacteria; 19 commonly identified only in pathogenic corynebacteria; and 50 only identified in *C. pseudotuberculosis*). Annotations were obtained following analyses with the Blast2GO tool [84], used through the web application available at http://www.blast2go.org/start_blast2go.

In total, 34 proteins identified in the exoproteome of the strain 1002 of *C. pseudotuberculosis *were found to be present in the experimentally determined extracellular proteomes of other corynebacteria, whereas the number of common corynebacterial exoproteins in the C231 strain was 32 (Figure 5). Only 6 proteins were consistently identified in all the corynebacterial exoproteomes, including pathogenic and non-pathogenic species: (i) S-layer protein A [62]; (ii) resuscitation-promoting factor RpfB [66]; (iii) cytochrome c oxidase subunit II [67]; (iv) a putative esterase; (v) a NLP/P60 family protein (putative cell wall-associated hydrolase) [68]; and (vi) a trehalose corynomycolyl transferase (Figure 5, additional file 8). Interestingly, three of these six proteins are predicted to be regulated by the same transcription factor [GenBank:ADL09702], a member of the cAMP receptor protein (Crp) family of transcription regulators which are found controlling a diversity of physiological functions in various bacteria [69].

Twelve proteins of the exoproteome of the 1002 strain and fifteen of the C231 strain were also detected experimentally only in the exoproteomes of other pathogenic corynebacteria, namely *C. diphtheriae *and *C. jeikeium *(Figure 5). Altogether, this represents 19 different *C. pseudotuberculosis *proteins (additional file 8). A search of similarity using the sequences of these proteins against publicly available databases, believed to contain the predicted proteomes of all corynebacteria with completely sequenced genomes, showed that 6 of these 19 proteins are apparently absent from non-pathogenic corynebacterial species (Table 1). Moreover, 5 of these proteins are predicted to be part of regulatory networks already shown to be involved in virulence functions, including those regulated by the diphtheria toxin repressor (DtxR)-like protein [70] and the cAMP-binding transcription regulator GlxR [71].

Two proteins presented orthologs highly distributed in various bacterial pathogens: (i) a putative iron transport system binding (secreted) protein [GenBank:ADL10460]; and (ii) a putative glycerophosphoryl diester phosphodiesterase [GenBank:ADL11410]. Interestingly, an ortholog of this latter protein was included recently in a list of seventeen proteins found to be very common in pathogenic bacteria and absent or very uncommon in non-pathogens, representing then probable virulence-associated factors [72]. In fact, reports in the literature can be found that associate orthologs of the two aforementioned proteins with virulence phenotypes [73,74]. Noteworthy, both proteins were detected in this study only in the exoproteome of the C231 strain of *C. pseudotuberculosis*, the more virulent one.

## Conclusions

There seems to be a growing interest in profiling the exoproteomes of bacterial pathogens, due to the distinguished roles played by exported proteins on host-pathogen interactions [10]. Classical proteomic profiling strategies, normally involving two-dimensional (2D) gel electrophoresis, have been extensively used for this purpose [16-20]. Nevertheless, the introduction of more high-throughput proteomic technologies brings new perspectives to the study of bacterial exoproteomes, as it makes it easier to analyze multiple phenotypically distinct strains, yielding better subproteome coverage with fewer concerns regarding technical sensitivity and reproducibility [75]. Besides, the currently available methods for label-free quantification of proteins [76] allow us to compare the "dynamic behavior" of the exoproteome across different bacterial strains, and this in turn will help us to better identify alterations of the exoproteome that may contribute to the various virulence phenotypes.

By using a high-throughput proteomic strategy, based on a recently introduced method of LC-MS acquisition (LC-MS^E^) [14], we were able to perform a very comprehensive analysis of the exoproteome of an important veterinary pathogen, *Corynebacterium pseudotuberculosis*. Comparative exoproteome analysis of two strains presenting different virulence status allowed us to detect considerable variations of the core *C. pseudotuberculosis *extracellular proteome, and thereby the number of exoproteins identified increased significantly. Most importantly, it was helpful to gain new insights into the probable participation of *C. pseudotuberculosis *exported proteins, other than the well-known PLD and FagB, in the virulence of this bacterium. Several novel targets for future work on *C. pseudotuberculosis *molecular determinants of virulence can be identified from the catalogue of exoproteins generated in this study. Interestingly, around 30% of the proteins identified were predicted by the SurfG+ software [15] as being probably surface exposed in *C. pseudotuberculosis*. Such proteins may represent promising new candidates for composing a CLA vaccine more effective than the ones currently available [4], as has been demonstrated for a series of other bacterial pathogens [33,34]. Therefore, it will be critical to further study the role of this protein set in virulence and vaccine design.

## Methods

### Bacterial strains and culture conditions

The strains 1002 and C231 of *Corynebacterium pseudotuberculosis *were used in this study. Strain 1002 was isolated from an infected goat in Brazil and has been shown to be naturally low virulent [23,56]; strain C231 was isolated from an infected sheep in Australia, and it showed a more virulent phenotype [24]. Species confirmation was performed by biochemical and molecular methods for both strains, as described [77]. Complete genome sequences of the two strains were generated by Genome Networks in Brazil and Australia (RGMG/RPGP and CSIRO Livestock Industries), and made available for this study (unpublished results).

*C. pseudotuberculosis *strains were routinely maintained in Brain Heart Infusion broth (BHI: Oxoid, Hampshire, UK) or in BHI 1.5% bacteriological agar plates, at 37°C. For proteomic studies, strains were grown in a chemically defined medium (CDM) previously optimized for *C. pseudotuberculosis *cultivation [78]. The composition of the CDM was as follows: autoclaved 0.067 M phosphate buffer [Na_2_HPO_4_**·**7H_2_O (12.93 g/L), KH_2_PO_4 _(2.55 g/L), NH_4_Cl (1 g/L), MgSO_4_**·**7H_2_O (0.20 g/L), CaCl_2 _(0.02 g/L), and 0.05% (v/v) Tween 80]; 4% (v/v) MEM Vitamins Solution 100X (Invitrogen); 1% (v/v) MEM Amino Acids Solution 50X (Invitrogen); 1% (v/v) MEM Non Essential Amino Acids Solution 100X (Invitrogen); and 1.2% (w/v) filter-sterilized glucose.

### Three-phase partitioning

Extraction/concentration of the soluble supernatant proteins of *C. pseudotuberculosis *followed the TPP protocol previously optimized by our group [11], with minor modifications. Briefly, overnight cultures (*ca*. 24 hours) of the different *C. pseudotuberculosis *strains were inoculated (1:100) separately into 500 mL of pre-warmed fresh CDM and incubated at 37°C, with agitation at 100 rpm, until reach the mid-exponential growth phase (OD_540 nm _= 0.4; LabSystems iEMS Absorbance Plate Reader). At this point, cultures were centrifuged at room temperature (RT) for 20 min, 4000 rpm, and 400 mL of each supernatant was transferred into new sterile flaks. Following addition of 20 μL Protease Inhibitor Cocktail P8465 (Sigma-Aldrich), supernatants were filtered through 0.22 μm filters; ammonium sulphate was added to the samples at 30% (w/v) and the pH of the mixtures were set to 4.0. Then, *n*-butanol was added to each sample at an equal volume; samples were vigorously vortexed and left to rest for 1 h at RT, until the mixtures separated into three phases. The interfacial precipitate was collected in 1.5 mL microtubes, and re-suspended in 1 mL Tris 20 mM + 10 μL protease inhibitor. Finally, samples were submitted to diafiltration and buffer exchange with NH_4_HCO_3 _(100 mM), using 5 kDa cut-off spin columns (Millipore).

### In-solution tryptic digestion of TPP-extracted proteins

Protein samples were resuspended in 1 mL of 0.1% Rapigest (Waters Corporation, Milford, MA) and concentrated using a 5 kDa cut-off spin column. The solution was heated at 80°C for 15 minutes, reduced with dithiothreitol, alkylated with iodoacetamide and digested with 1:50 (w/w) sequencing grade trypsin for 16 hours. RapiGest was hydrolysed by the addition of 2 μL of 13 M trifluoroacetic acid, filtered using a 0.22 μm spin column and each sample was typically diluted to 1 μg/μL prior to a 1:1 dilution with a 100 fmol/μL glycogen phosphorylase B standard tryptic digest to give a final protein concentration of 500 ng/μL per sample and 50 fmol/μL phosphorylase B.

### LC-MS configurations for label-free analysis (LC-MS^E^)

Nanoscale LC separations of tryptic peptides for qualitative and quantitative multiplexed LC-MS analysis were performed with a nanoACQUITY system (Waters Corporation) using a Symmetry C_18 _trapping column (180 μm × 20 mm 5 μm) and a BEH C_18 _analytical column (75 μm × 250 mm 1.7 μm). The composition of solvent A was 0.1% formic acid in water, and solvent B (0.1% formic acid in acetonitrile). Each sample (total digested protein 0.5 μg) was applied to the trapping column and flushed with 0.1% solvent B for 2 minutes at a flow rate of 15 μL/min. Sample elution was performed at a flow rate of 250 nL/min by increasing the organic solvent concentration from 3 to 40% B over 90 min. Three technical replicate injections of the TPP-extracted 1002 sample and four technical replicates of the TPP-extracted C231 sample were used for subsequent data analysis in this study. These were from two biological cultures of each *C. pseudotuberculosis *stain.

The precursor ion masses and associated fragment ion spectra of the tryptic peptides were mass measured with a Q-ToF Ultima Global or Synapt HDMS mass spectrometer (Waters Corporation) directly coupled to the chromatographic system. The time-of-flight analyzers of both mass spectrometers were externally calibrated using the MS/MS spectrum from [Glu^1^]-Fibrinopeptide B (human - Sigma Aldrich, UK) obtained from the doubly charged peptide ion at *m/z *785.8426. The monoisotopic mass of the doubly charged species in MS mode was also used for post-acquisition data correction. The latter was delivered at 500 fmol/μL to the mass spectrometer via a NanoLockSpray interface using the auxiliary pump of a nanoACQUITY system at a flow rate of 500 nL/min, sampled every 60 seconds.

Accurate mass data were collected in data independent mode of acquisition by alternating the energy applied to the collision cell/s between a low and elevated energy state (MS^E^). The spectral acquisition scan rate was typically 0.9 s with a 0.1 s interscan delay. On the Synapt HDMS instrument in the low energy MS mode, data were collected at constant trap and transfer collision energies (CE) of 3 eV and 1 eV respectively. In elevated energy MS mode, the trap collision energy was ramped from 15 eV to 30 eV with the transfer collision energy at 10 eV. On the Ultima Global instrument a low energy of 6 eV was applied to the collision cell, increasing from 6 eV to 35 eV in elevated MS mode.

### Data processing for label-free acquisitions (MS^E^)

The LC-MS^E ^data were processed using ProteinLynx Global Server v2.4 (Waters Corporation, Milford, MA) (see additional file 9). In brief, lockmass-corrected spectra are centroided, deisotoped, and charge-state-reduced to produce a single accurately mass measured monoisotopic mass for each peptide and the associated fragment ion. The initial correlation of a precursor and a potential fragment ion is achieved by means of time alignment. The detection and correlation principles for data independent, alternate scanning LC-MS^E ^data have been described [14].

### Database searches

All data were searched using PLGS v2.4 against a *Corynebacterium pseudotuberculosis *database (NCBI Genome Project ID: 40687 and 40875), released in November 2009, to which the glycogen phosphorylase B and trypsin sequences had been appended. The database was randomised within PLGS generating a new concatenated database consisting of the original sequences plus one additional sequence for each entry with identical composition but randomly scrambled residues. This database contained a total of 4314 entries. A fixed modification of carbamidomethyl-C was specified, and variable modifications included were acetyl N-terminus, deamidation N, deamidation Q and oxidation M. One missed trypsin cleavage site was permitted.

For the MS^E ^data, the time-based correlation applied in data processing is followed by a further correlation process during the database search that is based on the physicochemical properties of peptides when they undergo collision induced fragmentation. The precursor and fragment ion tolerances were determined automatically. The initial protein identification criteria used by the Identity^E ^algorithm within PLGS for a single replicate data file, required the detection of at least three fragment ions per peptide, seven fragment ions and a minimum of one peptide per protein.

A process analogous to the Bayesian model described by Nesvizhskii *et al*. [79] was used by PLGS to assign probability values to scores of peptide and protein identifications. Two automated mechanisms determined peptide and protein threshold identification criteria providing a 95% identification confidence interval. A background search is conducted by the search algorithm creating a discriminating decoy identification distribution. The determined peptide cut-off score, typically a log value of 6.25 for the expected 95% identification probability is automatically applied to the results.

Further more stringent filtering was then applied to the database search results from each sample to improve the confidence in the protein observations and quantitative measurements. The results from each of the *individual *replicate analyses from each sample were combined and proteins were removed that were observed in only one of the replicates. Using this additional and rigorous filter the false discovery rate was further reduced to 0.2% for this study, with an average of 16.5 peptides/protein and 37.5% sequence coverage for the TPP-extracted 1002 sample and 15 peptides/protein with 35% sequence coverage for the respective C231 sample. Proteins were observed on average in 2.81 technical replicates in the 1002 sample where 3 replicate analyses were used and 3.52 for the C231 sample in which 4 replicates were included.

### Protein quantification using label-free system (MS^E^)

Relative quantitative analysis between samples was performed by comparing normalized peak area/intensity of each identified peptide [80]. For relative quantification, automatic normalization was applied to the data set within PLGS using the total peptide complement of each sample. The redundant, proteotypic quantitative measurements generated from the tryptic peptide identifications from each protein were used to determine an average, relative protein fold-change, with a confidence interval and a regulation probability. The confidently identified peptides to protein ratios were automatically weighted based on their identification probability. Binary comparisons were conducted to generate an average normalized intensity ratio for all matched proteins. The entire data set of differentially expressed proteins was further filtered by considering only the identified proteins that replicated in at least two technical replicates with a score > 250 and likelihood of regulation value greater than 0.95 for upregulation and lower than 0.05 for downregulation as determined by the PLGS quantification algorithm.

### *In silico *predictions of protein sub-cellular localization

Prediction of sub-cellular localization was performed initially for the identified proteins by using the SurfG+ program v1.0, run locally in a Linux environment, as described [15] (see additional file 9). For prediction of potentially surface exposed (PSE) proteins, a cut-off value of 73 amino acids was calculated as the minimum distance from the *C. pseudotuberculosis *outermost membrane until the surface of the cell-wall, based on electron microscopy of this bacterium's cell envelope (data not shown).

The programs TatP v1.0 and SecretomeP v2.0 were used through the web applications available at http://www.cbs.dtu.dk/services/, for prediction of twin-arginine pathway-linked signal peptides and non-classical (leaderless) secretion, respectively [29,81].

### Comparative analyses of multiple corynebacterial exoproteomes

A list of experimentally observed extracellular proteins of pathogenic (*C. diphtheriae *and *C. jeikeium*) and non-pathogenic (*C. glutamicum *and *C. efficiens*) corynebacteria was identified in previously published studies [17,37,64,65]. The amino acid sequences of these proteins were retrieved from public repositories of protein sequences to create a local database. This database was used in similarity searches with the Blast-p algorithm (E-value < 10^-4^) [26], taking the group of proteins identified in the *C. pseudotuberculosis *exoproteome as the input sequences. Additionally, transitivity clustering [82] was used to identify proteins (i) commonly detected in the exoproteomes of pathogenic and non-pathogenic corynebacteria, and proteins detected in exoproteomes of (ii) only pathogenic corynebacteria or (iii) only *C. pseudotuberculosis*. A more detailed description on the transitivity clustering analysis can be found in the supplementary material (additional file 9). The amino acid sequences of the identified *C. pseudotuberculosis *exoproteins were also used in similarity searches against public databases, namely NCBI nr and Swissprot.

### Transcriptional regulation of the identified exoproteins

The search for transcription factors that regulate expression of the identified corynebacterial exoproteins was performed through the CoryneRegNet database, as described previously [83].

### Accession numbers

The sequences of all proteins identified in this work are accessible through GenBank and correspond to the *Corynebacterium pseudotuberculosis *Genome Projects deposited in NCBI (IDs: 40687 and 40875).

## List of abbreviations

CDM: chemically defined medium; CLA: caseous lymphadenitis; LC-MS: liquid chromatography - mass spectrometry; NCS: non-classically secreted; PLD: phospholipase D; PLGS: ProteinLynx Global Server; PMF: peptide mass fingerprinting; PSE: potentially surface exposed; RGMG: Minas Gerais Genome Network; RPGP: Genome and Proteome Network of the State of Pará; TPP: Three-Phase Partitioning.

## Competing interests

The authors declare that they have no competing interests.

## Authors' contributions

LGCP, SES, LMF, MARC, AMCP, RM, AS, JHS, SCO, AM, CGD, and VA conceived the idea, participated in the design of the study, and critically read the manuscript. LGCP, SES, NS, TLPC, WMS, AGV, and SGS performed microbiological and/or proteomic experiments. LGCP, SES and ARS performed bioinformatical analyses. LGCP and SES wrote the manuscript. All authors read and approved the final manuscript.

## Supplementary Material

Additional file 1 **Figure S1. Comparison between the experimental (A) and virtual (B) 2-D gels of the exoproteome of the strain 1002 of *C. pseudotuberculosis***. (A) 2D-gel with 150 μg of TPP extracted extracellular proteins of the 1002 strain. Proteins were separated in the first dimension by isoelectric focusing using strips of 3.0-5.6 NL pI range (GE Healthcare). Visualization was by Colloidal Coomassie staining. (B) The virtual 2D-gel was generated with the theoretical pI and MW values of the proteins identified by LC-MS^E^. Click here for file

Additional file 2 Table S1. Proteins composing the core *C. pseudotuberculosis *exoproteome, identified by LC-MS^E^. Click here for file

Additional file 3 Table S2. Variant exoproteome of the strain 1002 of *Corynebacterium pseudotuberculosis*. Click here for file

Additional file 4 Table S3. Variant exoproteome of the strain C231 of *Corynebacterium pseudotuberculosis*. Click here for file

Additional file 5 Figure S2. Predictions of LPXTG motif-containing proteins, lipoproteins and Tat-pathway associated signal peptides in the exoproteomes of the strains 1002 and C231 of *C. pseudotuberculosis*. Click here for file

Additional file 6 **Figure S4. A conserved hypothetical exported protein present in the Genome of the strain C231 but absent from the strain 1002 of *C. pseudotuberculosis***. The two sequenced Genomes were aligned using the Artemis Comparison Tool (ACT). The arrows point to tRNA genes. Click here for file

Additional file 7 Table S4. Relative expression analysis of the extracellular proteins common to the strains 1002 and C231 of *Corynebacterium pseudotuberculosis*. Click here for file

Additional file 8 **Figure S5. Distribution of orthologous proteins of the *C. pseudotuberculosis *experimental exoproteins throughout other experimentally confirmed exoproteomes of pathogenic corynebacteria, as determined through transitivity clustering analysis**. The 19 *C. pseudotuberculosis *exoproteins only identified in the exoproteomes of other pathogenic corynebacteria are presented in the table. *Cp *= *C. pseudotuberculosis*; *Cd *= *C. diphtheriae*; *Cj *= *C. jeikeium*. Click here for file

Additional file 9 Supplementary information on the bioinformatics tools used in this study. Click here for file
