# Supplementary figures and images for: A combined approach for comparative exoproteome analysis of Corynebacterium pseudotuberculosis
Source: BMC Microbiol. 2011 Jan 17;11:12. doi: 10.1186/1471-2180-11-12 (PMC3025830; doi:10.1186/1471-2180-11-12)

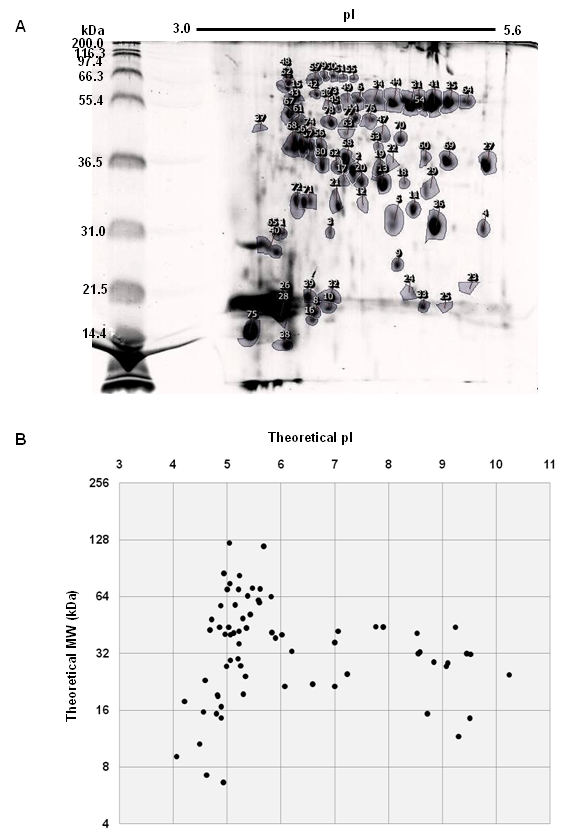

Supplement: Additional file 1 — Figure S1. Comparison between the experimental (A) and virtual (B) 2-D gels of the exoproteome of the strain 1002 of C. pseudotuberculosis. (A) 2D-gel with 150 μg of TPP extracted extracellular proteins of the 1002 strain. Proteins were separated in the first dimension by isoelectric focusing using strips of 3.0-5.6 NL pI range (GE Healthcare). Visualization was by Colloidal Coomassie staining. (B) The virtual 2D-gel was generated with the theoretical pI and MW values of the proteins identified by LC-MSE. [file 1471-2180-11-12-S1.TIFF]

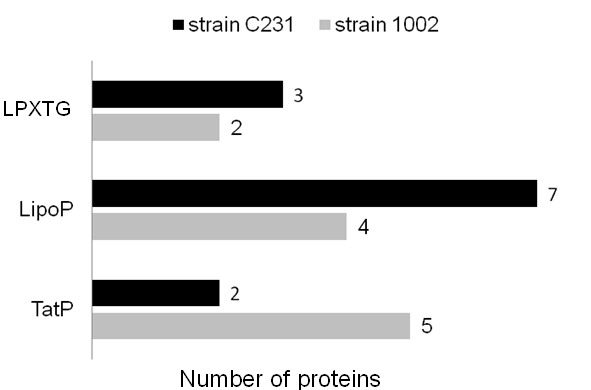

Supplement: Additional file 5 — Figure S2. Predictions of LPXTG motif-containing proteins, lipoproteins and Tat-pathway associated signal peptides in the exoproteomes of the strains 1002 and C231 of C. pseudotuberculosis. [file 1471-2180-11-12-S5.TIFF]

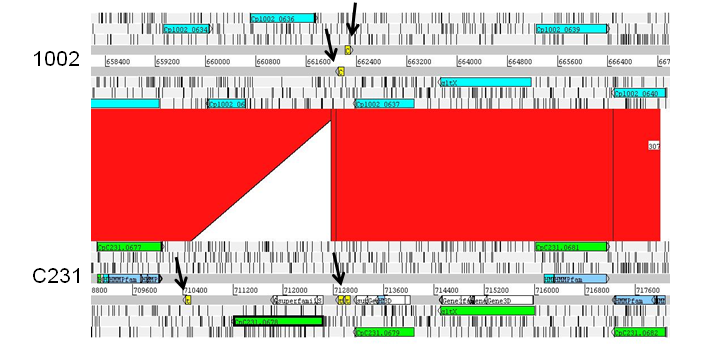

Supplement: Additional file 6 — Figure S4. A conserved hypothetical exported protein present in the Genome of the strain C231 but absent from the strain 1002 of C. pseudotuberculosis. The two sequenced Genomes were aligned using the Artemis Comparison Tool (ACT). The arrows point to tRNA genes. [file 1471-2180-11-12-S6.TIFF]

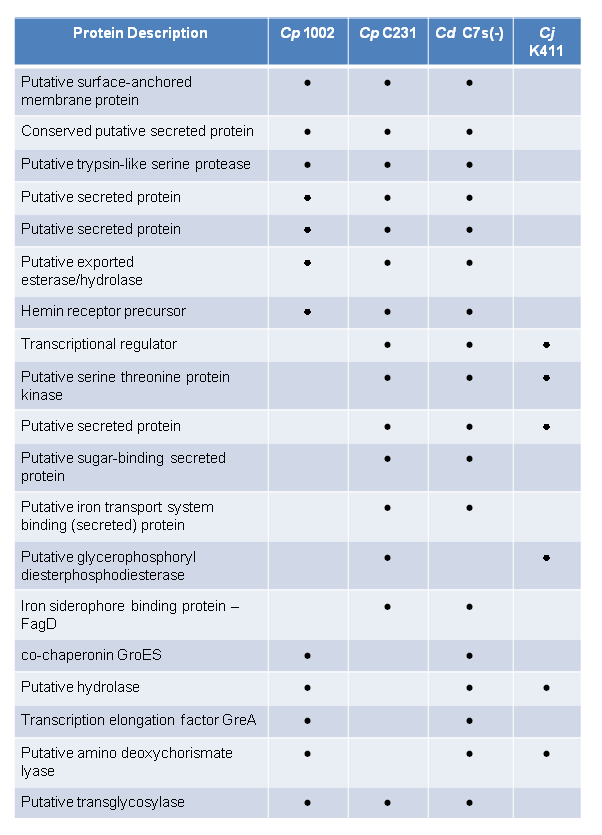

Supplement: Additional file 8 — Figure S5. Distribution of orthologous proteins of the C. pseudotuberculosis experimental exoproteins throughout other experimentally confirmed exoproteomes of pathogenic corynebacteria, as determined through transitivity clustering analysis. The 19 C. pseudotuberculosis exoproteins only identified in the exoproteomes of other pathogenic corynebacteria are presented in the table. Cp = C. pseudotuberculosis; Cd = C. diphtheriae; Cj = C. jeikeium. [file 1471-2180-11-12-S8.TIFF]
