# Supplementary material for: A combined approach for comparative exoproteome analysis of Corynebacterium pseudotuberculosis
Source: BMC Microbiol. 2011 Jan 17;11:12. doi: 10.1186/1471-2180-11-12 (PMC3025830; doi:10.1186/1471-2180-11-12)
Supplement: Additional file 2 — Table S1. Proteins composing the core C. pseudotuberculosis exoproteome, identified by LC-MSE. [file 1471-2180-11-12-S2.PDF]

Additional file 2: Table S1 – Proteins composing the core *C. pseudotuberculosis* exoproteome, identified by LC-MS^E

	Protein description	Protein ID [a] in strain:		M_r / pI [b]	Ortholog [c]		Predicted localization by SurfG+ [d]	Number of peptides observed[e]		Sequence coverage (%) [f]	
		1002	C231		Organism	E-value		1002	C231	1002	C231
1	Conserved hypothetical protein	ADL19922	ADL09511	17.81 / 4.21	*C. diphtheriae*	$1e^{-27}$	E (S)	19	5	34.5	27.5
2	Putative membrane protein	ADL21840	ADL11428	41.30 / 5.83	*C. diphtheriae*	$1e^{-36}$	E (PSE)	52	24	47.0	48.5
3	Conserved putative secreted protein	ADL20770	ADL10365	30.00 / 5.20	*C. diphtheriae*	$2e^{-61}$	E (S)	20	14	42.1	38.6
4	Cell-surface hemin receptor	ADL21841	ADL11429	71.00 / 5.47	*C. striatum*	$5e^{-101}$	E (PSE)	63	24	63.6	38.5
5	Putative cytochrome c oxidase subunit II	ADL21302	ADL10895	40.20 / 6.02	*C. diphtheriae*	0	E (PSE)	18	14	33.3	31.9
6	Putative cell-surface hemin receptor	ADL21338	ADL10936	84.15 / 5.40	*C. diphtheriae*	0	E (PSE)	48	31	49.9	41.9
7	Putative membrane protein	ADL20351	ADL09946	32.54 / 8.58	*C. diphtheriae*	$3e^{-69}$	E (PSE)	26	13	49.2	40.0
8	Conserved hypothetical protein	ADL21049	ADL10640	11.60 / 9.30	*C. pseudogenitali.*	$9e^{-05}$	M	3	2	34.8	28.8
9	Secreted protein NLP/P60 family	ADL21293	ADL10887	37.00 / 5.81	*C. diphtheriae*	$6e^{-110}$	E (S)	13	11	36.8	32.3
10	Trehalose corynomycolyl transferase	ADL21812	ADL11400	70.30 / 5.61	*C. diphtheriae*	0	E (S) §	43	27	53.3	37.8
11	Putative invasion protein	ADL10547	ADL10547	61.40 / 5.58	*C. diphtheriae*	0	E (S)	60	45	53.5	56.7
12	Putative HtaA family protein	ADL21337	ADL10935	32.00 / 8.55	*C. diphtheriae*	$7e^{-98}$	E (PSE)	30	19	42.6	48.4
13	NLP/P60 family secreted protein†	ADL21294	ADL10888	21.41 / 7.00	*C. diphtheriae*	$2e^{-91}$	E (S)	2	1	10.5	5.3
14	Substrate-binding protein	ADL21914	ADL11501	123.00/ 5.04	*C. striatum*	0	E (PSE) ¥	104	70	56.0	57.4
15	Hypothetical protein	ADL09626	ADL09626	24.27 / 9.24	*D. fermentans*	1.7	C * /E (S)	13	13	44.2	48.5
16	Resuscitation-promoting factor RpfA	ADL20487	ADL10080	21.42 / 6.07	*C. diphtheriae*	$5e^{-80}$	E (S)	11	8	37.5	36.2
17	Putative penicillin-binding protein	ADL21890	ADL11477	77.16 / 8.67	*C. diphtheriae*	0	E (S) / (PSE)	15	13	28.8	24.2
18	Putative secreted protein	ADL20288	ADL09881	27.33 / 9.07	*C. diphtheriae*	$4e^{-84}$	E (S)	14	9	49.0	37.2
19	Putative surface-anchored protein	ADL21911	ADL11498	90.60 / 5.10	*C. diphtheriae*	$6e^{-159}$	E (S) / (PSE)	40	42	48.2	52.5

20	Hypothetical protein	ADL20222	ADL09817	44.10 / 9.24	*C. diphtheriae*	$6e^{-12}$	E (PSE)	14	16	39.9	44.9
21	Putative secreted protein	ADL21925	ADL11512	14.56 / 4.89	*C. hylemonae*	0.34	E (S)	13	13	38.4	51.8
22	Putative exported esterase hydrolase	ADL20788	ADL10383	32.90 / 6.20	*C. diphtheriae*	$8e^{-76}$	C	8	7	26.7	21.5
23	Putative membrane protein	ADL21275	ADL10868	24.61/10.24	*C.diphtheriae*	$7e^{-54}$	E (PSE)	5	12	26.0	45.5
24	Putative heme transport associated protein	ADL20347	ADL09942	64.00 / 5.82	*C. ulcerans*	0	E (PSE)	23	24	39.7	42.4
25	Putative efflux system protein	ADL21747	ADL11333	59.60 / 5.60	*C. diphtheriae*	$2e^{-80}$	E (PSE)	26	25	50.3	44.4
26	Putative phosphatase	ADL09864	ADL09864	44.31 / 7.76	*C. jeikeium*	$2e^{-119}$	E (S)	12	14	30.0	29.6
27	Putative surface-anchored membrane protein	ADL20074	ADL09668	118.41/5.68	*C. diphtheriae*	0	E (PSE) ¥	37	52	33.6	43.8
28	Resuscitation-promoting factor RpfB	ADL20574	ADL10163	40.22 / 5.06	*C. diphtheriae*	$5e^{-142}$	E (S)	20	18	39.6	36.1
29	Putative secreted hydrolase	ADL20429	ADL10025	32.00 / 9.45	*C.lipophiloflavum*	$7e^{-54}$	E (S)	6	11	30.3	37.0
30	Trehalose corynomycolyl transferase	ADL21814	ADL11402	36.60 / 7.00	*C. diphtheriae*	$1e^{-133}$	E (S)	10	9	27.7	25.5
31	Putative secreted hydrolase	ADL20134	ADL09728	28.83 / 8.84	*C.aurimucosum*	$2e^{-64}$	E (S)	8	9	41.4	39.3
32	Surface layer protein A	ADL20140	ADL09734	38.60 /5.90	*C. diphtheriae*	$2e^{-141}$	E (S)	29	19	59.6	38.1
33	Putative secreted protein	ADL21714	ADL11301	42.00 / 5.22	*C. diphtheriae*	$4e^{-104}$	E (S)	28	26	51.5	56.5
34	Corynomycolyl transferase	ADL21610	ADL11196	42.00 / 7.06	*C. glutamicum*	$2e^{-103}$	E (S)	11	15	39.4	33.1
35	Putative sialidase precursor	ADL20287	ADL09880	75.00 / 5.05	*C. diphtheriae*	0	E (S)	42	29	48.6	41.6
36	Putative trypsin-like serine protease	ADL20653	ADL10245	28.43 / 9.10	*C. diphtheriae*	$9e^{-57}$	E (S)	10	7	33.8	26.7
37	Putative peptide transport system secreted protein	ADL20650	ADL10241	57.30 / 4.88	*C. diphtheriae*	0	E (PSE) +	11	14	25.9	29.4
38	Putative secreted protein	ADL20508	ADL10099	31.65 / 9.52	*C. diphtheriae*	$6e^{-96}$	E (S) §	3	6	13.9	20.7
39	Putative peptidoglycan recognition protein	ADL21828	ADL11416	70.00 / 5.00	*C. glutamicum*	$4e^{-164}$	E (S)	13	13	22.7	20.4
40	Secreted subtilisin-like peptidase	ADL21499	ADL11094	64.54 / 5.38	*D. nodosus*	$2e^{-95}$	E (S)	14	11	26.3	26.6

41	Putative peptidyl prolyl cis trans isomerase A	ADL19928	ADL09517	19.30 / 4.82	*C. diphtheriae*	$2e^{-86}$	C #	5	5	29.2	29.8
42	Hemin receptor precursor	ADL20348	ADL09943	40.50 / 4.96	*C. ulcerans*	$5e^{-175}$	E (PSE) +	12	9	34.2	37.5
43	Metalloendopeptidase-like protein	ADL20536	ADL10125	24.85 / 7.23	*C. diphtheriae*	$2e^{-91}$	E (PSE)	5	6	26.4	39.2
44	Putative serine protease	ADL20555	ADL10144	49.01 / 5.29	*C. diphtheriae*	$4e^{-141}$	C * / E (PSE)	6	10	18.9	22.6

Proteins above the thick line were considered to be differentially expressed in the two strains, according to the PLGS v2.4 quantification algorithm (see text and additional file 8: Table S4). Numbering of the proteins refers to Figure 3.

[a] Accession numbers in Entrez Protein (NCBI Genome Projects 40687 and 40875).

[b] Theoretical molecular weights (Mr) and isoelectric points (pI), calculated by the Compute pI/MW tool (ExPASY tools).

[c] Major similarity found by Blast-p against the nr database: *Corynebacterium diphtheriae; Corynebacterium ulcerans; Corynebacterium glutamicum; Corynebacterium striatum; Corynebacterium jeikeium; Corynebacterium aurimucosum; Corynebacterium lipophiloflavum; Corynebacterium pseudogenitalium; Dyadobacter fermentans; Clostridium hylemonae; Dichelobacter nodosus.*

[d] E = extracytoplasmic; S = secreted; PSE = potentially surface exposed; C = cytoplasmic; M = membrane.

[e, f] Average values calculated from three experimental replicates.

§ Predicted Tat-associated signal peptide.

+ Predicted lipoprotein.

¥ Predicted LPXTG cell wall-anchoring motif.

∗ SecretomeP prediction of non-classical secretion.

# Extensive literature evidence for exportation by non-classical pathways.

† Only this protein was identified by a single peptide in strain C231, but it was observed in strain 1002 with 2 peptides. These peptides were consistently identified in three technical replicates.
