# Supplementary material for: A combined approach for comparative exoproteome analysis of Corynebacterium pseudotuberculosis
Source: BMC Microbiol. 2011 Jan 17;11:12. doi: 10.1186/1471-2180-11-12 (PMC3025830; doi:10.1186/1471-2180-11-12)
Supplement: Additional file 3 — Table S2. Variant exoproteome of the strain 1002 of Corynebacterium pseudotuberculosis. [file 1471-2180-11-12-S3.PDF]

Additional file 3: Table S2 – Variant exoproteome of the strain 1002 of *Corynebacterium pseudotuberculosis*

Protein description	Protein ID [a]	M_r / pI [b]	Ortholog [c]		Predicted localization by SurfG+ [d]	Number of peptides observed[e]	Sequence coverage (%) [f]
			Organism	E-value			
Putative trypsin-like serine protease	ADL20466	41.00 / 8.53	*C. diphtheriae*	$2e^{-100}$	E (S) §	16	43.1
Conserved hypothetical protein	ADL21628	19.50 / 5.30	*C. glutamicum*	$4e^{-48}$	E (S) §	4	29.7
Putative secreted protein	ADL20097	51.45 / 5.43	*C. matruchotii*	$6e^{-127}$	E (S) §	18	37.4
Putative hydrolase	ADL19973	43.64 / 5.36	*C. diphtheriae*	$2e^{-120}$	E (S)	10	25.6
PAP2 phosphatase	ADL09864	44.21 / 7.90	*C. jeikeium*	$4e^{-117}$	E (S)	12	30.0
Putative secreted protein	ADL20656	24.20 / 5.34	*C. diphtheriae*	$5e^{-54}$	E (S)	5	30.8
Putative exported lipase	ADL21667	44.04 / 5.03	*C. diphtheriae*	$4e^{-126}$	E (S)	4	13.5
Putative secreted protein	ADL20455	22.00 / 6.59	*G. bronchialis*	$2e^{-06}$	E (S)	8	35.1
Putative amino deoxychorismate lyase	ADL21028	41.10 / 5.12	*C. diphtheriae*	$7e^{-165}$	E (S)	6	21.6
Putative secreted protein	ADL21239	23.07 / 4.59	*C. diphtheriae*	$6e^{-37}$	E (PSE) +	5	28.0
ABC superfamily ATP binding cassette transporter	ADL20218	57.80 / 5.15	*C. jeikeium*	$3e^{-159}$	E (PSE) +	15	34.8
co-chaperonin GroES	ADL20318	10.60 / 4.49	*C. diphtheriae*	$3e^{-48}$	C #	5	36.4
Phosphoglyceromutase	ADL20161	27.51 / 5.25	*C. diphtheriae*	$5e^{-125}$	C #	8	39.1
Elongation factor Tu	ADL20240	44.00 / 4.86	*C. diphtheriae*	0	C #	12	45.2
Elongation factor Ts	ADL21187	29.40 / 5.06	*C. diphtheriae*	$6e^{-131}$	C #	13	49.9
Transcription elongation factor GreA	ADL20612	19.01 / 4.83	*C. diphtheriae*	$1e^{-87}$	C *	8	43.7
Conserved hypothetical protein	ADL20222	15.35 / 8.72	*A.ferrooxidans*	1.2	C *	5	45.2
Peroxiredoxin	ADL21047	15.70 / 4.56	*C. glutamicum*	$3e^{-48}$	C #	2	24.7
Phosphoglycerate kinase	ADL20990	42.60 / 4.68	*C. diphtheriae*	0	C #	7	20.3
Triosephosphate isomerase	ADL20989	27.35 / 4.99	*C. diphtheriae*	$2e^{-121}$	C #	8	36.9
Conserved hypothetical protein	ADL21342	7.26 / 4.62	*C. diphtheriae*	$1e^{-19}$	C *	5	58.5
Putative carbohydrate carrier protein	ADL21114	9.1 / 4.06	*C. diphtheriae*	$1e^{-37}$	C	1†	14.8
FHA domain protein (OdhI)	ADL20881	15.34 / 4.80	*C. diphtheriae*	$2e^{-67}$	C	4	38.8
Putative methylmalonyl CoA	ADL20739	16.70 / 4.89	*C. diphtheriae*	$2e^{-75}$	C	4	37.2

epimerase							
Putative lipoprotein	ADL19972	48.50 / 4.71	*C. diphtheriae*	$3e^{-159}$	C	8	24.8
Hypothetical protein	ADL11253	6.64 / 4.93	No significant similarity found.	_	C	6	91.7

[a] Accession numbers in Entrez Protein (NCBI Genome Projects 40687 and 40875).
[b] Theoretical molecular weights (Mr) and isoelectric points (pI), calculated by the Compute pI/MW tool (ExPASY tools).
[c] Major similarity found by Blast-p against the nr database: *Corynebacterium diphtheriae; Corynebacterium matruchotii; Corynebacterium glutamicum; Corynebacterium jeikeium; Acidithiobacillus ferooxidans.*
[d] E = extracytoplasmic; S = secreted; PSE = potentially surface exposed; C = cytoplasmic; M = membrane.
[e, f] Average values calculated from three experimental replicates.
§ Predicted Tat-associated signal peptide.
+ Predicted lipoprotein.
¥ Predicted LPXTG cell wall-anchoring motif.
∗ SecretomeP prediction of non-classical secretion.
# Extensive literature evidence for exportation by non-classical pathways.
† This protein was consistently identified by a single peptide [(K) VTVTSENAEAVEK (I); observed m/z = 688.84].
