# Supplementary material for: A combined approach for comparative exoproteome analysis of Corynebacterium pseudotuberculosis
Source: BMC Microbiol. 2011 Jan 17;11:12. doi: 10.1186/1471-2180-11-12 (PMC3025830; doi:10.1186/1471-2180-11-12)
Supplement: Additional file 4 — Table S3. Variant exoproteome of the strain C231 of Corynebacterium pseudotuberculosis. [file 1471-2180-11-12-S4.PDF]

Additional file 4: Table S3 – Variant exoproteome of the strain C231 of *Corynebacterium pseudotuberculosis*

Protein description	Protein ID [a]	M_r / pI [b]	Ortholog [c]		Predicted localization by SurfG+ [d]	Number of peptides observed[e]	Sequence coverage (%) [f]
			Organism	E-value			
Phospholipase D (PLD)	ADL09524	32.45 / 8.77	*C.pseudotuberculosis*	$4e^{-173}$	E (S)	11	33.6
Serine proteinase precursor (CP40)	ADL11339	43.00 / 6.48	*C.pseudotuberculosis*	0	E (S)	25	48.4
Hypothetical protein	ADL10001	27.21 / 4.77	*C.diphtheriae*	$2e^{-06}$	E (S)	7	32.9
Putative secreted protein	ADL09871	16.80 / 8.30	*C. tropicalis*	0.87	E (S)	6	34.6
Conserved hypothetical exported protein	ADL10384	49.61 / 8.69	*C.diphtheriae*	$7e^{-96}$	E (S)	9	19.0
Hypothetical protein	ADL11213	33.55 / 4.48	*C.diphtheriae*	$6e^{-08}$	E (S)	12	32.7
Putative secreted protein	ADL21537	23.4 / 9.36	*C.litoralis*	1.2	E (S)	7	43.7
Putative secreted protein	ADL10489	24.73 / 5.07	*C.diphtheriae*	$9e^{-77}$	E (S)	7	37.7
Secreted penicillin-binding protein	ADL09532	50.75 / 5.67	*C.diphtheriae*	0	E (S)	12	29.7
Putative secreted protein	ADL21555	26.57 / 5.43	*C.diphtheriae*	$1e^{-74}$	E (S)	7	30.3
Putative surface-anchored protein	ADL11344	27.85 / 9.24	*C. jeikeium*	$9e^{-21}$	E (S)	5	17.2
Putative penicillin-binding secreted protein	ADL09697	83.00 / 5.38	*C.diphtheriae*	0	E (S)	14	22.2
Putative membrane anchored protein	ADL11338	21.15 / 9.59	*C.diphtheriae*	$6e^{-16}$	E (S)	6	19.1
Hypothetical protein	ADL11326	34.65 / 5.85	*H.mukohataei*	$8e^{-07}$	E (PSE)	13	36.4
Transcriptional regulator	ADL09990	55.05 / 4.97	*C.diphtheriae*	0	E (PSE)	13	38.5
Putative serine threonine protein kinase	ADL10880	80.10 / 4.80	*C.diphtheriae*	0	E (PSE)	10	16.2
Putative extracellular solute-binding protein	ADL09852	61.54 / 5.31	*C.glucuronolyticum*	0	E (PSE)	13	30.7
Maltotriose-binding protein	ADL09872	43.93 / 5.07	*C.diphtheriae*	$5e^{-180}$	E (PSE) +	10	30.4
Putative zinc metallopeptidase	ADL10626	35.38 / 5.29	*C.diphtheriae*	$3e^{-110}$	E (PSE)	7	25.1

Putative iron transport system binding (secreted) protein	ADL10460	30.05 / 5.16	*C.diphtheriae*	$8e^{-119}$	E (PSE) +	6	25.2
Glycerophosphoryl diesterphosphodiesterase	ADL11410	40.28 / 5.13	*C. amycolatum*	$2e^{-113}$	E (PSE) +	10	32.2
Putative metal-binding like protein	ADL10663	20.81 / 5.48	*C.diphtheriae*	$4e^{-65}$	E (PSE) +	10	51.2
Iron siderophore binding protein – FagD	ADL09528	37.47 / 5.05	*C.pseudotuberculosis*	0	E (PSE) +	5	16.3

[a] Accession numbers in Entrez Protein (NCBI Genome Projects 40687 and 40875).
[b] Theoretical molecular weights (Mr) and isoelectric points (pI), calculated by the Compute pI/MW tool (ExPASY tools).
[c] Major similarity found by Blast-p against the nr database: *Corynebacterium diphtheriae; Corynebacterium glutamicum; Corynebacterium amycolatum; Candida tropicalis; Congregibacter litoralis; Halomicrobium mukohataei.*
[d] E = extracytoplasmic; S = secreted; PSE = potentially surface exposed; C = cytoplasmic; M = membrane.
[e, f] Average values calculated from three experimental replicates.
§ Predicted Tat-associated signal peptide.
+ Predicted lipoprotein.
¥ Predicted LPXTG cell wall-anchoring motif.
∗ SecretomeP prediction of non-classical secretion.
Extensive literature evidence for exportation by non-classical pathways.
