# Supplementary material for: A combined approach for comparative exoproteome analysis of Corynebacterium pseudotuberculosis
Source: BMC Microbiol. 2011 Jan 17;11:12. doi: 10.1186/1471-2180-11-12 (PMC3025830; doi:10.1186/1471-2180-11-12)
Supplement: Additional file 7 — Table S4. Relative expression analysis of the extracellular proteins common to the strains 1002 and C231 of Corynebacterium pseudotuberculosis. [file 1471-2180-11-12-S7.PDF]

Additional file 7: Table S4 – Relative expression analysis of the extracellular proteins common to the strains 1002 and C231 of *Corynebacterium pseudotuberculosis*.

	Protein ID		PLGS Score	1002:C231 Log(e)Ratio	1002:C231 Log(e)StdDev
1	ADL19922	ADL09511	3334.63	1.49	0.14
2	ADL21840	ADL11428	3866.6	1.28	0.13
3	ADL20770	ADL10365	2567.58	1.09	0.13
4	ADL21841	ADL11429	3213.03	0.73	0.09
5	ADL21302	ADL10895	1976.92	0.61	0.25
6	ADL21338	ADL10936	1629.57	0.59	0.15
7	ADL20351	ADL09946	7055.8	0.57	0.13
8	ADL21049	ADL10640	2905.33	0.45	0.22
9	ADL21293	ADL10887	431.41	0.4	0.21
10	ADL21812	ADL11400	2125.62	0.23	0.1
11	ADL10547	ADL10547	7901.5	0.11	0.08
12	ADL21337	ADL10935	6080.98	-0.25	0.11
13	ADL21294	ADL10888	2326.18	-0.28	0.17
14	ADL21914	ADL11501	6925.86	-0.31	0.15
15	ADL09626	ADL09626	32362.36	-0.31	0.09
16	ADL20487	ADL10080	10299.72	-0.35	0.14
17	ADL21890	ADL11477	250.83	-0.55	0.58
18	ADL20288	ADL09881	6375.42	-0.57	0.16
19	ADL21911	ADL11498	12286.3	-1	0.2
20	ADL20222	ADL09817	586.53	-1.09	0.39
21	ADL21925	ADL11512	45622.2	-1.18	0.1
22	ADL20788	ADL10383	1168.04	-1.22	0.52
23	ADL21275	ADL10868	1078.49	-1.26	0.9
24	ADL20347	ADL09942	2801.18	-1.32	0.17
25	ADL21747	ADL11333	3829.92	-1.34	0.17
26	ADL09864	ADL09864	1240.67	-1.43	0.59
27	ADL20074	ADL09668	3420.41	-1.65	0.16
28	ADL20574	ADL10163	10163.62	-1.8	0.09
29	ADL20429	ADL10025	2076.33	-1.84	0.4
30	ADL21814	ADL11402	6280.63	-1.92	0.25
31	ADL20134	ADL09728	1411.1	-1.98	0.25
32	ADL20140	ADL09734	4999.15	-2.08	0.11
33	ADL21714	ADL11301	11768.53	-2.17	0.16
34	ADL21610	ADL11196	1858.86	-2.27	0.24
35	ADL20287	ADL09880	4203.95	-0.04	0.09
36	ADL20653	ADL10245	2062.52	0.14	0.16
37	ADL20650	ADL10241	215.41	0.33	0.32
38	ADL20508	ADL10099	234.37	0.15	0.37

39	ADL21828	ADL11416	208.26	-0.91	0.42
40	ADL21499	ADL11094	171.94	-0.34	0.52
41	ADL19928	ADL09517	109.49	-0.37	0.77
42	ADL20348	ADL09943	150.32	-1.04	0.98
43	ADL20536	ADL10125	557.8	-0.25	0.59
44	ADL20555	ADL10144	199.59	-1.05	0.95

Proteins above the thick line were considered to be differentially expressed in the two strains, according to the PLGS v2.4 quantification algorithm. Numbering of the proteins refers to Table 1 and Figure 3.
