# Supplementary material for: A combined approach for comparative exoproteome analysis of Corynebacterium pseudotuberculosis
Source: BMC Microbiol. 2011 Jan 17;11:12. doi: 10.1186/1471-2180-11-12 (PMC3025830; doi:10.1186/1471-2180-11-12)
Supplement: Additional file 9 — Supplementary information on the bioinformatics tools used in this study. [file 1471-2180-11-12-S9.PDF]

Additional file 9 – Supplementary information on the bioinformatics tools used in this study

Transitivity clustering

Transitivity clustering analysis was performed using the TransClust tool with Java Web Start (available at: http://transclust.cebitec.uni-bielefeld.de/) (. A dataset containing the amino acid sequences of 370 corynebacterial exoproteins, experimentally identified in five different proteomic studies (see main article text for details), was submitted to a all-vs.-all Blast-p analysis using an E-value cutoff of 10^-5. This multiple alignment yielded a tabular result file that was employed along with the FASTA file of protein sequences in transitivity clustering. The analysis demonstrated that the threshold rendering the best density of clusters for this dataset was 185. Then, novel executions of the clusterization algorithm were performed, using thresholds between 0-185, with 0.3 intervals, rendering hundreds of possibilities of clusters. To elect the "best threshold", a subset of 44 different proteins commonly identified previously in the two *C. pseudotuberculosis* strains by the ProteinLynx Global Sever software was elected as a positive control group. So, we looked for the threshold that would generate the same 44 groups out of a total of 137 (93 distinct) *C. pseudotuberculosis* proteins experimentally identified in the exoproteomes of the two strains studied (refer to Figure 1 in the main article, and Table S1). The best threshold for transitivity clustering analysis in this study was defined as 49. In total, 118 clusters were generated from the group of 370 corynebacterial exoproteins. Using this threshold, our results were slightly improved, compared to the previous results obtained only with Blast-p analysis: one novel protein was identified that was present in all corynebacterial exoproteomes studied (before there were 5 proteins in this group, and now there are 6); and one protein, before believed to be unique of the *C. pseudotuberculosis* exoproteome, was included in the group of proteins identified only in pathogenic corynebacteria (before there were 18 proteins in this group, and now there are 19).

ProteinLynx Global Server v2.4

ProteinLynx Global Server™ (PLGS) (Waters Corporation, Milford, MA) is an integrated platform for qualitative and quantitative studies using Waters™ proteomics systems. It is the only analytical platform that supports the MS^E mode of data-independent MS acquisition. The $Identity^E$ and $Expression^E$ algorithms feature XML based informatics (mzData supported) for accurate protein identification and label-free quantification (absolute or relative) (more information available from: http://www.waters.com/).

Protein identification and quantification were performed in this study with PLGS as described in details in recent technical publications (Silva *et al.*, 2005; Silva *et al.*, 2006; Geromanos *et al.*, 2009), using an Electrospray-MS^E template created as per the ProteinLynx Global SERVER User´s Guide (freely available upon subscription at http://www.waters.com/).

SurfG+ analysis

In silico prediction of the sub-cellular localization of the *C. pseudotuberculosis* proteins was performed by the SurfG+ tool, run under Linux on a conventional PC, as described in details in the recent publication by Barinov and cols. (2009). A FASTA file containing the entire *C. pseudotuberculosis* predicted proteome was used as input.

References

1. Wittkop T, Emig D, Lange SJ, Rahmann S, Albrecht M, Morris JH, Boecker S, Stoye J, Baumbach J. **Partitioning biological data with Transitivity Clustering**. *Nature Methods* 2010, 7(6):419-20.

2. Silva JC, Gorenstein MV, Li G, Vissers JPC, Geromanos SJ: **Absolute quantification of proteins by LCMS^{E}: a virtue of parallel MS acquisition.** *Mol Cell Proteomics* 2006, 5:144-156.

3. Silva JC, Denny R, Dorschel CA, Gorenstein M, Kass IJ, Li G, McKenna T, Nold MJ, Richardson K, Young P, Geromanos S: **Quantitative proteomic analysis by accurate mass retention time pairs.** *Anal Chem* 2005, 77:2187-2200.

4. Geromanos SJ, Vissers JPC, Silva JC, Dorschel CA, Li G, Gorenstein MV, Bateman RH, Langridge JI: **The detection, correlation, and comparison of peptide precursor and product ions from data independent LC-MS with data dependant LC-MS/MS.** *Proteomics* 2009, 9:1683-1695.

5. Barinov A, Loux V, Hammani A, Nicolas P, Langella P, Ehrlich D, Maguin E, van de Guchte M: **Prediction of surface exposed proteins in *Streptococcus pyogenes*, with a potential application to other Gram-positive bacteria.** *Proteomics* 2009, 9:61-73.
